# Detection of supergroup B *Wolbachia* strains and their co-infection with *Plasmodium falciparum* in wild *Anopheles funestus* in southeastern Tanzania: implications for malaria control

**DOI:** 10.1186/s13071-026-07330-3

**Published:** 2026-03-17

**Authors:** Reuben E. Mmweteni, Anitha B. Philbert, Gustav Mkandawile, Faraji Abilahi, Saidi Abbasi, Jamal Msemo, Salum Milonge, Francis A. Tumbo, Yohana Mwalugelo, Letus L. Muyaga, Dickson Msaky, Abdallah R. Kipekepeke, Fredros O. Okumu, Emmanuel W. Kaindoa, Francesco Baldini

**Affiliations:** 1 College of Natural and Applied Sciences, Department of Zoology and Wildlife Conservation, University of Dar Es Salaam, P.O Box 35064, Dar Es Salaam, Tanzania; 2 Environmental Health and Ecological Sciences Department, Ifakara Health Institute, P. O. Box 53, Ifakara, Tanzania; 3 School of Biodiversity, One Health, and Veterinary Medicine, University of Glasgow, Glasgow, UK; 4 School of Life Sciences and Bioengineering, Nelson Mandela African Institute of Science and Technology, P.O Box 447, Arusha, Tanzania; 5 Wits Research Institute for Malaria, School of Pathology, Faculty of Health Sciences, University of the Witwatersrand and the Centre for Emerging Zoonotic and Parasitic Diseases, National Institute for Communicable Diseases, Johannesburg, South Africa

**Keywords:** *Wolbachia*, *Plasmodium falciparum*, *Anopheles funestus*, Polymerase chain reaction, Ifakara Health institute, Southeastern Tanzania

## Abstract

**Background:**

Evidence of natural infection with *Wolbachia* and its negative correlation with *Plasmodium falciparum* among wild malaria vectors has opened new avenues for utilization of *Wolbachia* in malaria vector control. However, the interaction between *Wolbachia* and *Plasmodium* parasites in mosquitoes tends to be species-specific and may show ecological variations. Among the primary malaria vectors in Tanzania, natural *Wolbachia* infection has only been observed in *Anopheles arabiensis*, while there is still limited information on *Wolbachia* natural infection in *Anopheles funestus* sensu lato, and its interaction with *P. falciparum* in the mosquito species. Therefore, this study investigated the prevalence of natural infection and co-infection of *Wolbachia* and *P. falciparum* in the *An. funestus* s.l. in southeastern Tanzania, and characterized the *Wolbachia* strains detected.

**Methods:**

The study was conducted in five villages in southeastern Tanzania between March and June 2024. Mosquitoes were collected from 52 households using Centers for Disease Control and Prevention (CDC) light traps and Prokopack aspirators, followed by morphological identification. Detection of *An. funestus* sibling species and *Wolbachia* was performed using conventional polymerase chain reaction (PCR) and nested PCR (*Wolbachia* only). Sanger sequencing was performed as a confirmatory test followed by phylogenetic analysis of the detected *Wolbachia* strains. *P. falciparum* sporozoites were detected using enzyme-linked immunosorbent assay (ELISA).

**Results:**

*Wolbachia* was detected in almost half of all wild *An. funestus* s.l. tested using the primary PCR (prevalence = 46.5%, *N* = 400); and more than half when nested PCR approach was used (prevalence = 70.8%, *N* = 400). Only three mosquitoes carried *P. falciparum* sporozoites (prevalence = 0.8%, *N* = 400) and only one showed co-infection with *Wolbachia* (prevalence = 0.3%, *n* = 400). Sequencing and phylogenetic analysis involving both the 16S rDNA, *coxA*, and *wsp Wolbachia* genes showed that the detected strains clustered with *Wolbachia* supergroup B, specific for Dipterans.

**Conclusions:**

Unlike findings from the previous study, this study demonstrates that *An. funestus* s.l. in southeastern Tanzania are infected with *Wolbachia*, at a surprisingly high prevalence. This study also provides the first report on *Wolbachia*–*P. falciparum* co-infection status in *An. funestus* s.l. in Tanzania. Further studies with larger sample sizes are needed to confirm the association between native *Wolbachia* and *P. falciparum* in wild *An. funestus* s.l. in southeastern Tanzania.

**Graphical Abstract:**

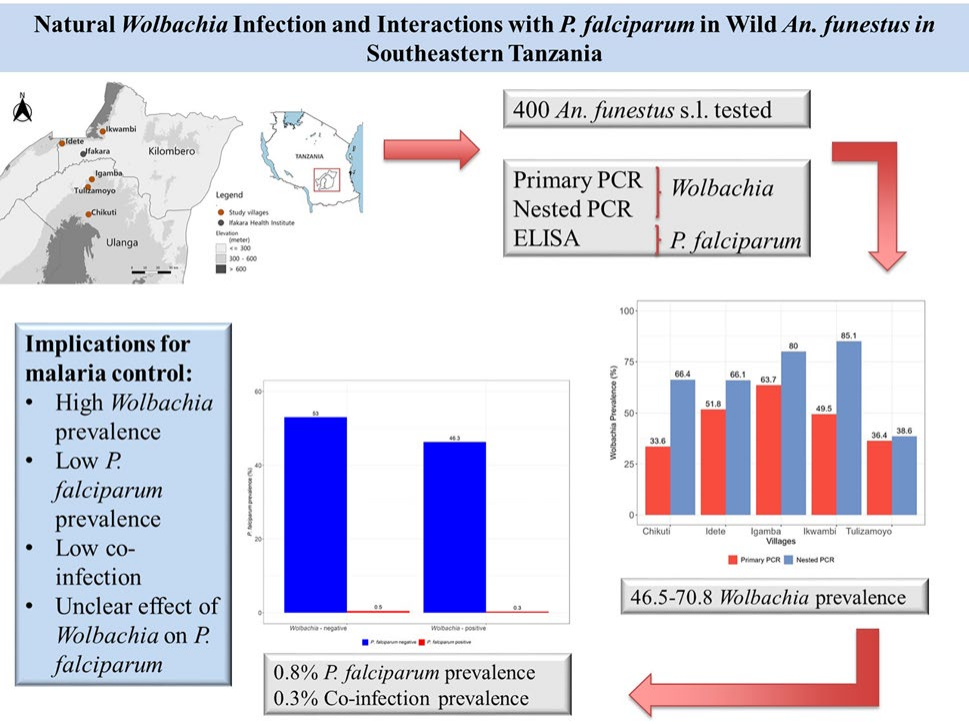

## Background

Malaria, a protozoan disease endemic in sub-Saharan Africa, is one of the world’s diseases of major public health concern [1, 2]. The disease can be spread by about 70 [3] out of more than 400 known species of the *Anopheles* genus, acting as primary or secondary vectors [4, 5]. However, only a few species are considered as efficient malaria vectors in sub-Saharan Africa, including *Anopheles funestus* s.s., *Anopheles gambiae* complex (particularly *Anopheles arabiensis*, *Anopheles coluzzii*, and *Anopheles gambiae*), *Anopheles moucheti*, and *Anopheles nili,* capable of transmitting both *Plasmodium falciparum* and *Plasmodium vivax* [4, 6, 7]. Controlling malaria vectors, by interfering with the *Plasmodium* parasite’s life cycle, has been a targeted approach by which to control malaria globally [5].

Malaria vector control, conventionally, uses tools such as long-lasting insecticidal nets, (LLINs) and indoor residue spraying (IRS) for controlling endophagic and endophilic malaria vectors as core interventions and control of the vector’s aquatic stages by larviciding as a supplementary intervention [8, 9]. Notwithstanding, the reliability of chemical-based control of malaria vectors is limited by the increasing resistance of the vectors to conventional insecticides, particularly pyrethroids, and mosquitoes’ behavioral avoidance of the LLINs and IRS by feeding and resting outdoors [8, 10–13].

One novel, promising biological approach to target malaria vectors both indoors and outdoors might involve the use of *Wolbachia*, a Gram-negative alpha-proteobacterium of the order Rickettsiales [14], first described a century ago as a Rickettsia-like microorganism [15]. *Wolbachia* genus is divided into 17 phylogenetic subclades or supergroups (named A–F, H–Q, and S); supergroups A and B are the most common among arthropods [14, 16, 17]. *Wolbachia* genus is further divided into strains, conventionally named after the host species they infect [18]. Different *Wolbachia* strains exhibit a range of symbiotic relationships with their hosts, limited to invertebrates, from parasitism to mutualism [19]. One such relationship is cytoplasmic incompatibility, where *Wolbachia* modifies the insect’s sperm, making it incompatible with the egg’s cytoplasm of *Wolbachia* uninfected females, resulting into nonviable offspring. This phenotype can be exploited to suppress disease-vector populations by releasing *Wolbachia*-infected males into *Wolbachia*-free populations. [14, 20–24]. One other relationship is *Wolbachia*-induced refractoriness of pathogens [25, 26], achieved by two possible mechanisms: *Wolbachia*-mediated competition of the host resources [27, 28] and/or *Wolbachia*-mediated upregulation of the host immune system [29]. Lab-based transinfection studies have shown that *Wolbachia* can protect *Aedes aegypti* from dengue, chikungunya, and yellow fever viruses and an avian malaria parasite, *P. gallinaecium*, which can infect *Ae. aegypti* in the lab [27, 30, 31]. In another study by Moretti et al. [32], *Wolbachia*-induced sterility and pathogen inhibition can be combined for population replacement and suppression of *Ae. albopictus*. In malaria vectors, there is some evidence for *Wolbachia*-induced parasite protection, however, this is not as strong as in *Aedes* [33, 34].

While the detection of natural *Wolbachia* infection in wild *Culex pipiens* and later in wild *Aedes albopictus* dates back to a century ago [15, 35, 36], natural *Wolbachia* infection in *Anopheles* mosquitoes was first reported in *Anopheles gambiae* s.l. in Burkina Faso only a decade ago [37]. Subsequently, reports on natural *Wolbachia* infection among wild *Anopheles* mosquitoes in various parts of sub-Saharan Africa were given [3, 37–42]. Indeed, Baldini et al. [37] noted that past failure to detect *Wolbachia* in anophelines, and thus the hypothesis that *Anopheles* mosquitoes are not *Wolbachia* infected, could have been methodologically rooted. Detection of native *Wolbachia* in *Anopheles* mosquitoes, particularly among the dominant malaria vectors in sub-Saharan Africa, sheds some light on preexistence of long-term interaction between the endosymbiont, malaria vectors, and malaria parasites. A subsequent report from Burkina Faso by Shaw et al. in 2016 [51] showed low prevalence of co-infection between *P. falciparum* and *Wolbachia* (*w*Anga) in *An. coluzzii*, highlighting that *w*Anga-infected *An. coluzzii* were less infected with *P. falciparum* infection than *w*Anga-uninfected mosquitoes. Similar findings of negative association between *w*Anga and *P. falciparum* were later reported in Mali [38]. Nonetheless, studies on natural *Wolbachia* infection in wild avian malaria vectors (*Simulium* genus) show that the correlation between *Wolbachia* and the malaria parasite (*Leucocytozoon*) is highly variable (negative, positive, or neutral) and is vector species specific [43]. This varying nature of interaction between *Wolbachia*, malaria parasites, and malaria vectors in field settings highlights the need for localized studies to elucidate desirable interactions for malaria control. Equally, it is important to characterize the dominant *Wolbachia* strains in wild populations of the malaria vectors so that the success of *Wolbachia*-based malaria vector control may not be constrained by the preexisting native *Wolbachia* infection [44].

The study conducted in one village in southeastern Tanzania detected two *Wolbachia* strains (referred to as *w*Anga_TZ) from *Wolbachia* supergroups A and B in *An. arabiensis* mosquitoes in 2014 and 2016. However, *Wolbachia* was not detected in *An. funestus* s.l. in the same study, and this was attributed to the small mosquito sample size tested [42]. Given that natural *Wolbachia* infection in *An. funestus* has already been documented in other regions [39, 40], and also considering the vector’s significant role in Tanzania’s malaria transmission [13, 45], it is important to conduct further investigation on natural *Wolbachia* infection in *An. funestus* s.l. in Tanzania with an increased sample size and a wider spatial coverage. This study investigated the prevalence of natural infection and co-infection of *Wolbachia* and *P. falciparum* in the *An. funestus* s.l. in southeastern Tanzania, and analyzed the phylogeny of the *Wolbachia* strains detected.

## Methods

### Study site

**Figure 1:**
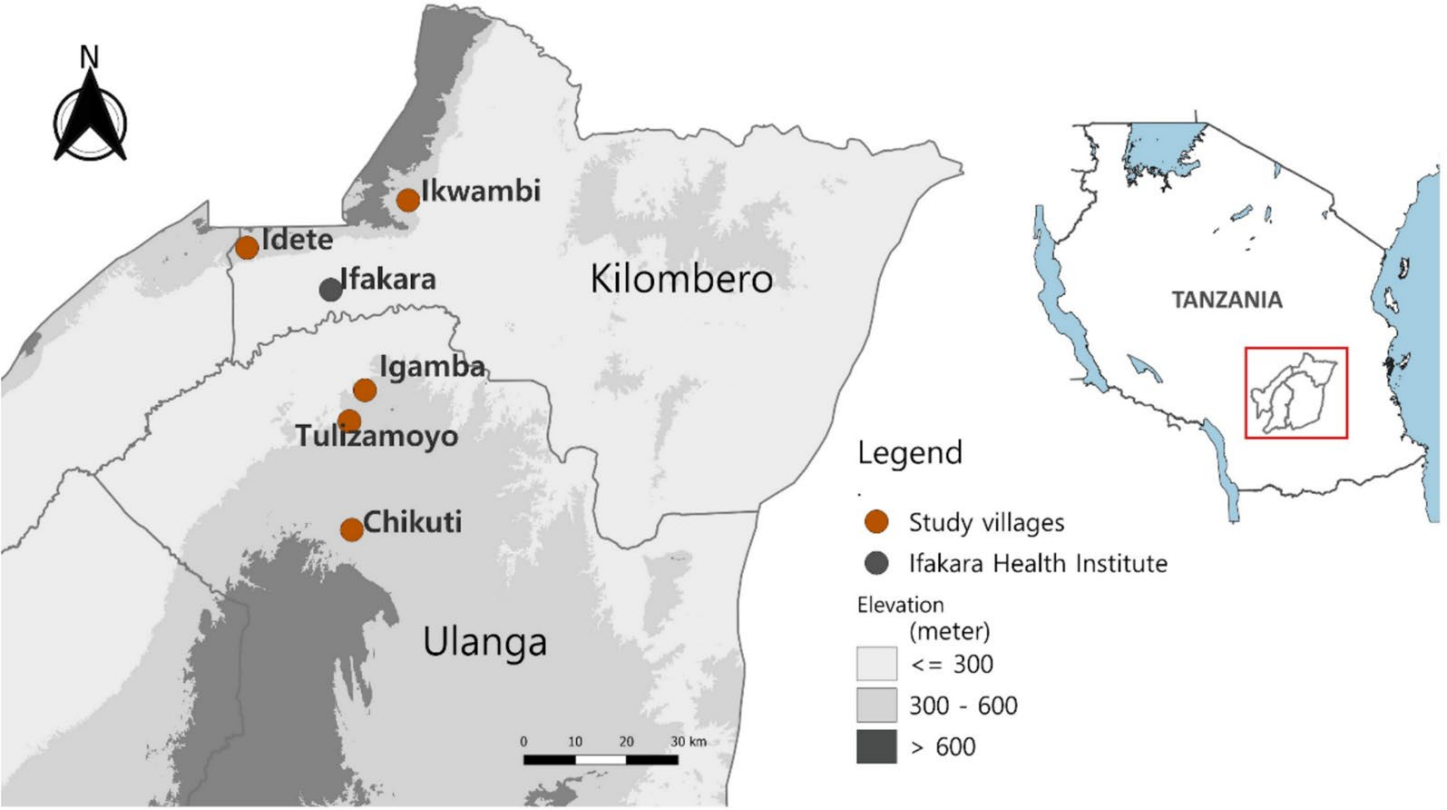
The study villages (Ikwambi, Idete, Igamba, Tulizamoyo, and Chikuti) were selected on the basis of the contribution of *An. funestus* s.l. in the area’s malaria transmission [43]. All collected, alive, *An. funestus* s.l. mosquitoes were transported to the Ifakara Health Institute laboratory for further analysis

This study was conducted in five villages from two districts in southeastern Tanzania; Tulizamoyo, Igamba, and Chikuti villages from Ulanga district (8.3124°S, 36.6879°E), and Ikwambi and Idete villages from Kilombero district (8.1539°S, 36.6870°E) (Fig. 1). The districts lie within the Kilombero valley, whose geospatial characteristics favor malaria transmission [46]. Similarly, *An. funestus* s.l. plays a significant role in the area’s malaria transmission [45].

#### Indoor mosquito collection, sorting, and identification

Host seeking and resting mosquitoes were trapped indoors by Centers for Disease Control and Prevention (CDC) light traps (John Hock, 7409 NW 23RD Ave, Gainesville, FL, 32,606 USA) between 18:00 and 06:00 h, and by Prokopack aspirator (Prevention, C.f.D.C.a., Model 1412, John Hock) around 06:00 h, respectively, from 52 households in the 5 study villages in southeastern Tanzania. Each village was visited twice but no household was visited more than once. The households were selected on the basis of accessibility (due to possible flooding as the study was conducted during the rainy season) and obtaining informed consent from the head of the household. All mosquitoes collected were sorted and morphologically identified using published identification keys [47, 48] and species quantification was restricted to *An. funestus* s.l. A few alive *Culex pipiens* complex, collected from Tulizamoyo village using CDC light trap, were selected and used for testing the study protocol. All *An. funestus* s.l. collected alive in the traps from all study villages were parked in net-covered paper cups and transported to the Ifakara Health Institute laboratory for further analysis. Other mosquito species identified (*An. gambiae* s.l. and *Culex* spp.) and all *An. funestus* s.l. found already dead in the traps were discarded from the study.

### Identification of *An. funestus* sibling species and *P. falciparum* sporozoite detection

Tris-EDTA (TE) buffer was used for deoxyribonucleic acid (DNA) extraction for sibling species identification of *An. funestus* s.l. using individual mosquitoes’ legs. All 400 mosquito DNAs were assessed by polymerase chain reaction (PCR) following the protocol established by Koekemoer et al. [49]. Enzyme-linked immunosorbent assay (ELISA) was performed to detect *P. falciparum* sporozoites using homogenates from head and thorax of individual mosquitoes. All ELISA lysates were boiled at 100 °C on a heating block for 10 min before the boiled-retest phase to eliminate false positives caused by heat unstable cross-reactive proteins [50].

### Detection of *Wolbachia* in *An. funestus* s.l. using *Wolbachia*-specific 16S ribosomal DNA (rDNA) primers

The DNA for *Wolbachia* detection was extracted from individual abdomens of fresh (just killed) mosquitoes using DNAzol kit (Thermo Scientific, US) followed by elution in 40 μl of nuclease free water. Extracted DNA from *Culex pipiens* complex was used for testing of the study protocol adopted from Shaw et al. [51], and were subsequently used as positive controls. *Wolbachia*-specific 16S rDNA primers [sequence; forward (FWD) 5′-CATACCTATTCGAAGGGATAG-3′, and reverse (REV) 5′-AGCTTCGAGTGAAACCAATTC-3′] [51] were used to amplify the 16S rDNA region of the *Wolbachia* DNA. Protocol adjustments were made on the final primer concentration in the master mix by halving the original primer concentration (0.35 mM) in the master mix and increasing PCR cycles from 35 to 40. The master mix was prepared by mixing [10 µl OneTaq, 0.28 µl of 10 µM (forward and reverse primers), and 5.44 µl DNAse free water] followed by 4 µl of DNA (final volume = 20 µl). The PCR was run under the following condition: 2 min period at 95 °C, two cycles of 2 min at 95 °C, 1 min at 60 °C, and 1 min at 72 °C, followed by four cycles of 30 s at 95 °C, 1 min at 60 °C, and 45 s at 72 °C and a post-dwell period of 5 min at 72 °C. Nested PCR approach established by Shaw et al. [51], considered a confirmatory test in this study, was done using *Wolbachia*-specific 16S rDNA primers (sequences; FWD 16SN- 5′GAA GGG ATA GGG TCG GTT CG-3′ and REV 16SN- 5′CAA TTC CCA TGG CGT GAC G-3′). Master Mix was prepared by mixing (10 µl OneTaq, 0.56 µl of 10 µM forward and reverse primers, and 6.88 µl DNAse free water), followed by 2 µl of the PCR product (final volume = 20 µl). The PCR was run under the following conditions: activation at 95 °C for 15 min, followed by 35 cycles of 95 °C for 15 s, 66 °C for 25 s, and 72 °C for 30 s, and a post-dwell period of 30 s at 72 °C. Both the first PCR (henceforth named primary PCR) and nested PCR were run in GeneAmp – PCR System 9700 machines.

### Detection of *Wolbachia* in *An. funestus* s.l. using *wsp* and *coxA* primers

For further confirmation of true *Wolbachia* infection, seven DNA samples showing strong *Wolbachia* diagnostic bands with *Wolbachia*-specific 16S rDNA primers were selected and tested for *Wolbachia* infection with *wsp* primers (sequences; forward *wsp* 81F 5′-TGG TCC AAT AAG TGA TGA AGA AAC-3′ and reverse *wsp* 691R 5′-AAA AAT TAA ACG CTA CTC CA-3′) and *coxA* primers (sequences; forward *coxA*_F1 5′-TTG GRG CRA TYA ACT TTA TAG-3′ and reverse *coxA*_R1 5′-CTA AAG ACT TTK ACR CCA GT-3′). A total of 4 μl of each DNA sample was added into the master mix of (10 µl OneTaq, 2 μl of 10 µM forward and reverse *wsp* primers, and 2 µl DNAse free water) making a final volume of 20 µl and into another master mix of 10 µl OneTaq, 2 μl of 10 µM forward and reverse *coxA* primers, and 2 µl DNAse free water, making a final volume of 20 µl. The PCR was run under the following conditions: 2-min period at 94 °C, followed by 37 cycles of 30 s at 94 °C, 45 s at 54 °C (*coxA*) or 59 °C (*wsp*), and 1 min 30 s at 72 °C, and a post-dwell period of 10 min at 72 °C, then 4 °C hold.

### Gel electrophoresis

The PCR products for *Wolbachia* detection and mosquito species identification were loaded on 1% and 2.5% agarose, respectively, followed by gel electrophoresis. GEL LOGIC 100 IMAGING SYSTEM and KODAK MI SE were used for gel imaging and display, respectively.

### Phylogenetic analysis

All *Wolbachia* sequences (16S rDNA, *coxA*, and *wsp*) were aligned using MUSCLE in MEGA 12. Reference 16S rDNA *Wolbachia* sequences representing members of supergroups A and B as used in Baldini et al. [42] were included to create a 16S rDNA-based *Wolbachia* tree. Reference *coxA Wolbachia* sequences representing members of supergroups A and B (supergroup A: *w*El OR227583.1, *w*Hm-a LC363924.1, *w*Hm-b LC363925.1; supergroup B: *w*Hm-c LC363926.1, *w*Bol MG920062.1, *w*Rr KJ620006.1, *w*Dec MK033289.1) were included to create a *coxA*-based *Wolbachia* tree. In addition, *Wolbachia* sequences representing members of supergroups A, B, D, and F (supergroup A: *w*Cne3 GU013552.1, *w*De AJ276615.1, *w*Es AB109621.1, *w*Pg AF521152; supergroup B: *w*Dec MK033272.1, *w*Scar KF378603.1, *w*Cne2 GU013551.1, *w*Dali KJ659909.1, *w*Pip OQ236547.1; supergroup D: *w*Bm OQ714005.1, *w*Wb DQ093846.1; supergroup F: *w*Cl AJ833930.1) were included as reference for creating the *wsp*-based *Wolbachia* tree. The sequences CP032049.1, KY114936.1, and NR_074500.2 for *Rickettsia japonica*, *Anaplasma phagocytophilum*, and *Ehrlichia chaffeensis*, respectively, and MT009148.1 for *Rickettsia amblyommatis*, were used as outgroups for the 16S rDNA and *coxA Wolbachia* phylogenetic analysis, respectively. Sequence divergences were calculated using the general time revisable (GTR + G) model, and subsequently, a maximum likelihood tree was created using 1000 bootstrap replicates of GTR + G distances as in Baldini et al. [42].

### Statistical analyses

All data were double entered and cleaned in Excel and analyzed using open source software, R version 4.5.1 [52]. The prevalence of *Wolbachia* and *P. falciparum* infection in *An. funestus* s.l. was calculated descriptively as the ratio of positive individuals to all mosquitoes tested × 100. Expected *Wolbachia* and *P. falciparum* co-infection prevalence was calculated as the prevalence of *Wolbachia* infection (prevalence estimated by nested PCR) times the prevalence of *P. falciparum* infection in *An. funestus* s.l. [38]. The observed co-infection prevalence was calculated as the number of *An. funestus* s.l. infected by both *P. falciparum* and *Wolbachia* in all mosquitoes screened × 100. Generalized linear mixed effect model with binomial distribution was used to analyze the difference in *Wolbachia* prevalence in *An. funestus* s.l. between primary and nested PCR; whereby PCR methods were the fixed variables, prevalence was the outcome variable, and villages, households, and mosquito collection rounds were the random variables. Nested models were compared with likelihood ratio tests.

## Results

### Identification of *An. funestus* sibling species

A total of 418 mosquitoes collected from all study villages were morphologically identified as *An. funestus* s.l., and only 400 that were alive during sorting were carried forward for laboratory analysis. Following PCR and gel electrophoresis, 397 mosquitoes were confirmed to be *An. funestus* s.s. (505 bp), while two mosquitoes amplified as *An. rivulorum* (410 bp) and *An. leesoni* (146 bp), respectively, and one mosquito amplified as a hybrid of *An. funestus* s.s. (505 bp) and *An. leesoni* (146 bp), even after repeating the assay for this sample.

#### Wolbachia detection using Wolbachia-specific 16S rDNA primers

**Figure 2:**
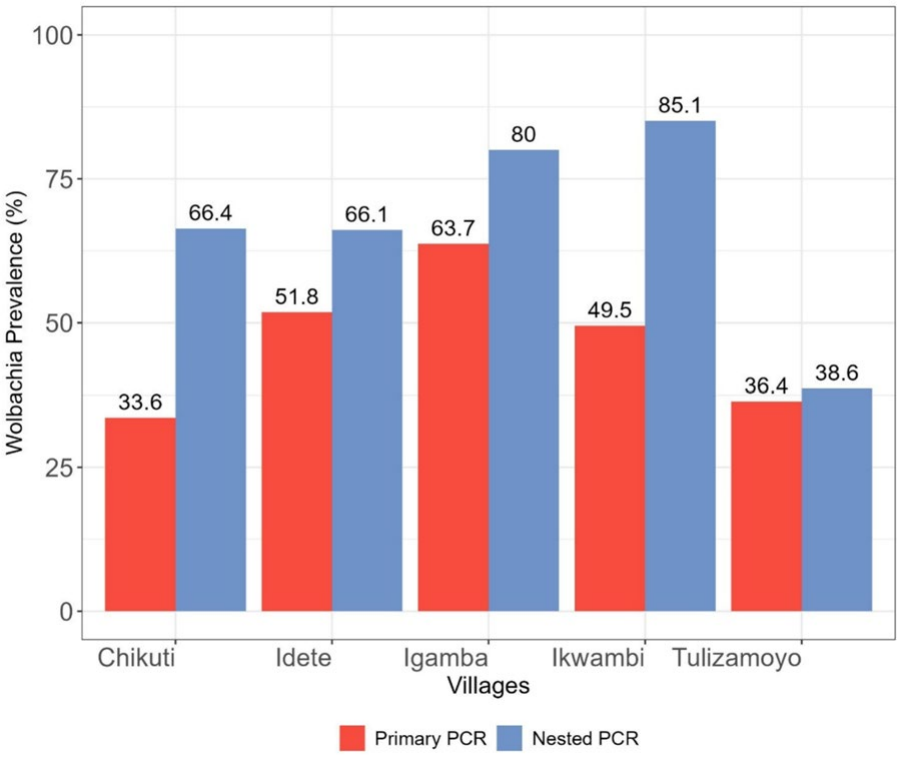
Prevalence of *Wolbachia* in *An. funestus* s.l. per village according to the method of PCR used

A total of 186 samples amplified as *Wolbachia* in the primary PCR (prevalence = 46.5%, *N* = 400), while 283 samples amplified as *Wolbachia* in the nested PCR (prevalence = 70.8%, *N* = 400). The prevalence of *Wolbachia* infection in *An. funestus* s.l. per village following the primary PCR ranged from 33.6% (*N* = 119) lowest prevalence in Chikuti Village to 63.8% (*N* = 80) highest prevalence in Igamba village (Fig. 2). An increase in *Wolbachia* prevalence with nested PCR was observed in every village; Tulizamoyo village had the lowest prevalence of 38.6% (*N* = 44) and Ikwambi village had the highest prevalence of 85.2% (*N* = 101). The overall prevalence of *Wolbachia* infection in *An. funestus* s.l. using nested PCR [prevalence = 70.8% (*N* = 400)], a 24.3% increase from the overall prevalence observed in the primary PCR (Table 1), was significantly higher than that observed in primary PCR [OR = 3.1 (95% CI 2.27–4.2, *P* < 0.001)].

**Table 1:** Comparison of two *Wolbachia* detection PCR methods

PCR method	*N*^a^	*W* + ^b^	Predictive mean	95% CI	OR^c^	95% CI	*Z*-value	*P*-value
Primary	400	186	0.9	0.42–2.0	1			
Nested	400	283	2.8	1.28–6.1	3.1	2.27–4.2	7.159	8.12 × 10^−13^

^a^Total number of mosquitoes tested

^b^*Wolbachia* positive

^c^Odds ratio

### *Wolbachia* detection using *wsp* and *coxA* primers

**Figure 3:**
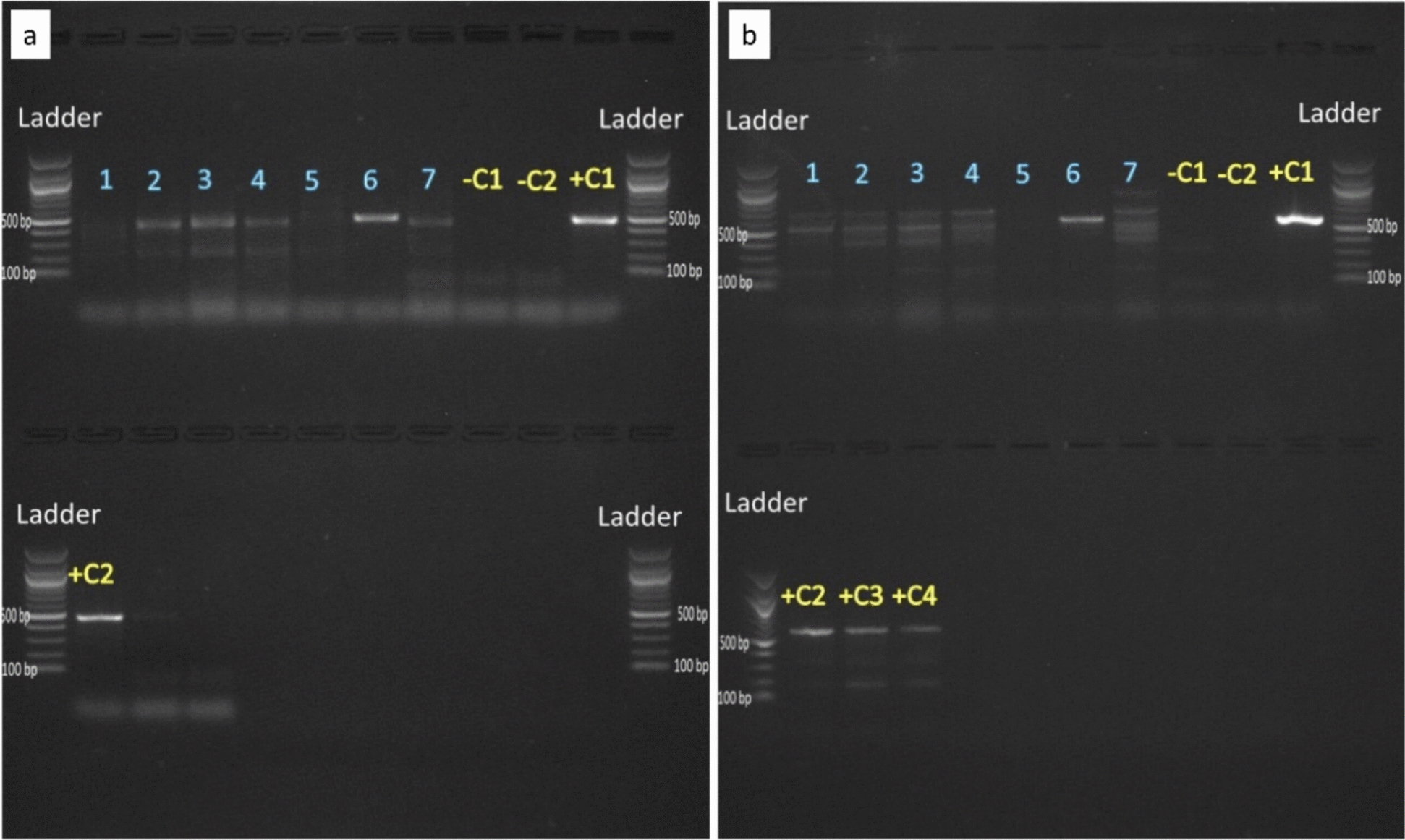
Photos of gel electrophoresis of *Wolbachia* samples amplified with **a** *coxA* and **b** *wsp* primers

Among the samples with highest amplification using 16S rDNA, we selected seven for further amplification and sequencing of *coxA*- and *wsp Wolbachia*-specific genes. Four out of seven samples amplified as *Wolbachia* using both *coxA* and *wsp* primers (Fig. 3a, b respectively), however, only one sample returned full sequences.

### Detection of *P. falciparum* sporozoites in *An. funestus* s.l.

Out of 400 *An. funestus* s.l. screened, only 3 *An. funestus* s.s. carried *P. falciparum* sporozoites, giving a prevalence of 0.8%. Two of the *An. funestus* s.s. infected with *P. falciparum* sporozoites were collected from Igamba village and one from Tulizamoyo village.

### Co-infection of *Wolbachia* and *P. falciparum* in *An. funestus* s.l.

**Figure 4:**
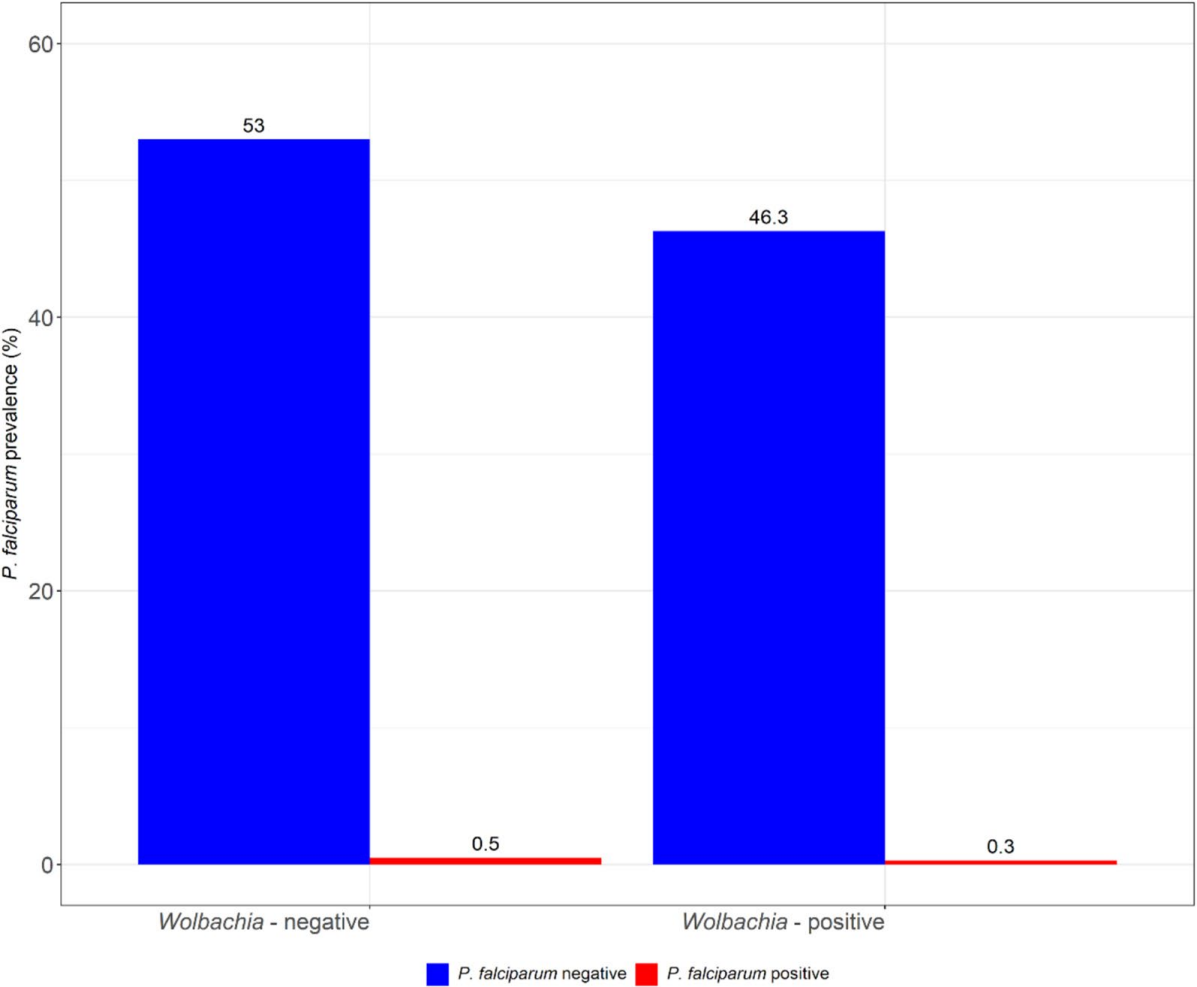
Low prevalence of *P. falciparum* infection (red) was observed in both groups, *Wolbachia*-infected and uninfected *An. funestus* s.l. Most *An. funestus* s.l., both *Wolbachia* infected and uninfected, were *P. falciparum* free (blue)

Two of the *P. falciparum-*positive *An. funestus* s.s. were not infected with *Wolbachia* using either primary or nested PCRs. Only one *An. funestus* s.s. showed co-infection with *P. falciparum* and *Wolbachia* using both PCR methods (prevalence = 0.3%, *N* = 400). The expected prevalence of *Wolbachia* and *P. falciparum* co-infection with primary and nested PCR was 0.4% and 0.5%, respectively, which is (1.3 and 1.7 times higher, respectively) than the observed co-infection prevalence. Figure 4 shows the co-infection prevalence of *Wolbachia* and *P. falciparum* sporozoites in *An. funestus* s.l. in southeastern Tanzania.

### Sequencing and phylogenetic analysis of *Wolbachia*

**Figure 5:**
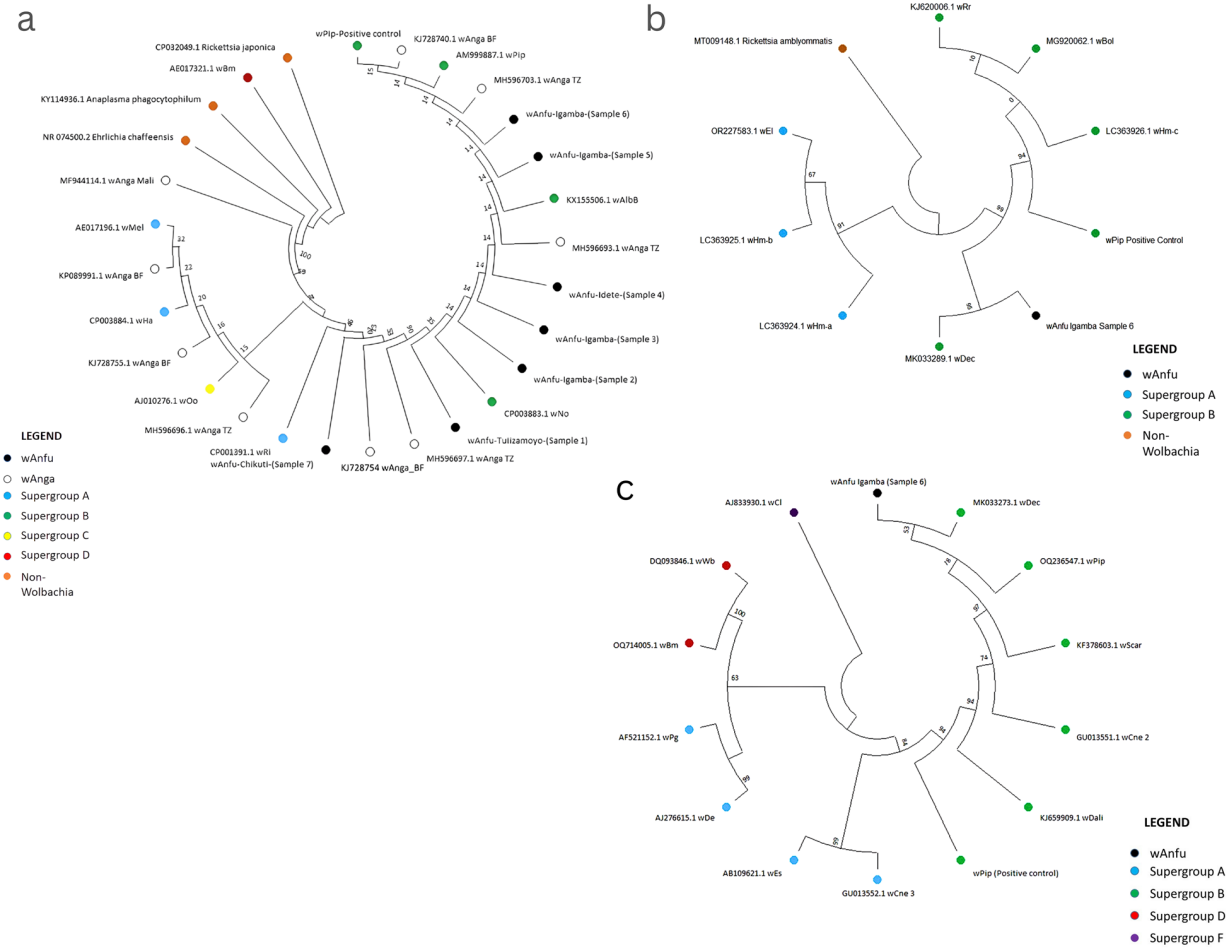
Phylogenetic analysis of *Wolbachia*-specific16S rDNA (**a**), *coxA* (**b**), and *wsp* (**c**) regions showing the detected *Wolbachia* strain clustering with supergroup B *Wolbachia* strains. *Wolbachia* supergroups A and B reference and outgroup sequences are included in (Figure 5**a**), as in Baldini et al. [41]. *Wolbachia* sequences for *Enoplagnatha latimana* and *Homona magnanima* strain a and b (*wEl*, *wHm*-a, and *wHm*-b) supergroup A; and *Homona magnanima* strain c, *Hypolimnas bolina, Rhinusa rara*, and *Culex decens (wHm*-c, *wBol*, *wRr*, and *wDec*) supergroup B are included in (Figure 5**b**). *Wolbachia* sequences for *Conotrachelus nenuphar* strain 3. *Dysdera erythrina*, *Elasmucha signoreti*, and *Pegoscapus gemellus* (*wCne* 3, *wDe*, *wEs*, and *wPg*) supergroup A; *Culex decens*, *Sciothrips cardamomi, Conotrachelus nenuphar* strain 2, *Bombyx mandarina* (Dali), and *Culex pipiens* (*wDec*, *wScar*, *wCne* 3, *w*Dali, and *wPip*) supergroup B; *Brugia malayi* and *Wuchereria bancrofti* (*wBm* and *wWb*) supergroup D; and *Coptotermes lacteus* (*wCl*) supergroup F are included in (Figure 5**c**)

All samples sequenced for the 16S rDNA corresponded to *Wolbachia*, and one sample (which amplified positively in *coxA* and *wsp* PCR) corresponded to *Wolbachia* after sequencing both the 16S rDNA, *coxA*, and *wsp Wolbachia* genes. Phylogenetic analysis involving both the 16S rDNA, *coxA*, and *wsp Wolbachia* genes showed that the detected *Wolbachia* strain clustered with the supergroup B *Wolbachia* strains, as did *w*Pip, the positive control (Fig. 5).

## Discussion

This study provides the first report on *Wolbachia* detection in wild *An. funestus* s.l., a competent malaria vector in Tanzania. The study increased both the sample size of *An. funestus* s.l. and the spatial coverage of the study area, unlike the previous study [42], and indeed, this is reflected in the high *Wolbachia* prevalence observed. The prevalence of *Wolbachia* infection in wild *An. funestus* s.l. in southeastern Tanzania [prevalence = 46.5% (primary PCR) and 70.8% (nested PCR), *N* = 400] is up to 9.4 times higher compared with that reported in *An. arabiensis* in the same area, using the same approach [42]. Despite the heterogeneity in the number of mosquito catches per village, which we attributed to the reduced mosquito abundance due to heavy rainfalls, the prevalence of *Wolbachia* infection in the mosquitoes per village was still significantly higher compared with that reported in the same species in other areas [39, 40].

The last decade has observed dynamics in the trend of *Wolbachia* detection among wild *Anopheles* mosquitoes from no detection at all, even among several important malaria vectors [53], up to 100% prevalence of *Wolbachia* detection [44]. The varying prevalence of *Wolbachia* detection among *Anopheles* species may be due to, but not limited to environmental, genetic, and host-specific factors [44, 54, 55]. Host-specific factors such as the interference of vertical transmission of *Wolbachia* by *Asaia*, a native microbiota of *Anopheles* mosquitoes including *An. funestus* s.l. [40, 56, 57] may influence the observation of low *Wolbachia* prevalence among the mosquitoes. This leaves questions on *Asaia* infection status in *An. funestus* s.l. and *An. arabiensis* in southeastern Tanzania, given the differences in *Wolbachia* prevalence observed between the two malaria vectors. Furthermore, insecticide resistance in *Culex pipiens* has been observed to increase their susceptibility to *Wolbachia* infection [58–61], calling for further exploration on the role of insecticide resistance in the dynamics of *Wolbachia* susceptibility in the two dominant malaria vectors, *An. arabiensis* and *An. funestus* s.l., in southeastern Tanzania. Similarly, genetic diversity, even among mosquitoes of the same species, temperature fluctuations, and mosquito age may influence the susceptibility of certain mosquito populations to *Wolbachia* infections [54, 62, 63]. Thermal stress, in particular, may have implications in the reduced and increased natural *Wolbachia* infection rates in wild mosquitoes [64]. Neither this study nor the previous one in the same area [42] considered the age of the *An. funestus* or *An. arabiensis* samples tested (this study did not include *An. arabiensis*), although this can influence *Wolbachia* prevalence [62, 65, 66]. Collection of *An. funestus* s.l. in both this study and the previous one in the same area [42], though a decade apart, was done during the rainy season, indicating that the mosquitoes were subject to more or less similar temperature conditions, and that factors other than temperature may be implicated in the observed differences in *Wolbachia* prevalence between the two studies. Nonetheless, the surprisingly high *Wolbachia* prevalence detected in the *An. funestus* s.l. in southeastern Tanzania provides a glimpse of the potential of the mosquito species to harbor stable *Wolbachia* infection in field setting.

Genotyping of the *Wolbachia* genus using the 16S rDNA, *wsp* gene, and the multilocus sequence typing (MLST) system targeting five conserved *Wolbachia* genes (*coxA, gatB, fbpA, hcpA, ftsZ*) is recommended to capture the diversity of *Wolbachia* strains [37, 38, 51, 67]. In this study, only the *coxA* from the MLST genes and *wsp* and the *Wolbachia*-specific 16S rDNA genes were used to genotype *Wolbachia*, and successful amplification of the genes using PCR and sequencing indicated true *Wolbachia* symbiosis in *An. funestus* s.l. in southeastern Tanzania [55]. Indeed, phylogenetic analysis of both the 16S rDNA, *coxA*, and *wsp Wolbachia* genes further confirmed the PCR and sequencing results, as the detected *Wolbachia* showed evolutionary relationship with supergroup B *Wolbachia* strains. Unlike the strain *w*Anga_TZ, detected in *An. arabiensis* in the same region, which showed clustering with *Wolbachia* from both supergroups A and B [42], the strain detected in this study only clusters with supergroup B *Wolbachia* strains, with an astounding consistency observed in both genes (16S rDNA, *coxA*, and *wsp*). The newly detected *Wolbachia* strains cluster separately from the positive control (*w*Pip), ruling out the possibility of contamination.

This study observed a very low prevalence of *P. falciparum* sporozoites in *An. funestus* s.s. alone (prevalence = 0.8%, *N* = 400). Other *An. funestus* sibling species—*An. rivulorum*, *An. leesoni*, and the possible hybrid of *An. funestus* s.s. and *An. leesoni*—detected in this study did not carry *P. falciparum* sporozoites. Different studies conducted in southeastern Tanzania and other malaria-endemic zones in different years have reported varying *P. falciparum* sporozoite prevalence in malaria vectors, ranging from 0% to as high as 9% [13, 45, 68–70]. Although this study did not assess the sporozoite rate in other malaria vectors, particularly *An. arabiensis*, various studies have shown that *An. funestus* s.s. tend to have higher sporozoite rate compared with *An. arabiensis*, thus primarily contributing to malaria transmission in southeastern Tanzania [13, 45, 68]. In contrast, *An. funestus* s.s. in this study were found to have very low sporozoite rate, unlike what was observed by Mapua et al. [70].

Given the low prevalence of *P. falciparum* sporozoite infection relative to *Wolbachia* in *An. funestus* s.l. observed in this study, a corresponding low rate of co-infection was anticipated. Indeed, the observed co-infection prevalence was 1.3 (primary PCR) and 1.7 (nested PCR) times lower than expected. Nevertheless, the low *P. falciparum* infection rate may be partly attributable to the limited sample size, which constrains the ability to assess any correlation between *Wolbachia* infection and sporozoite rates. In contrast, a similar study in Mali, which analyzed a much larger sample (*N* = 13,321), was able to establish a negative association between *Wolbachia* (*w*Anga_Mali) and *P. falciparum* infection in *An. gambiae* s.l., with *Wolbachia*-infected females less likely to harbor *P. falciparum* than uninfected ones [38]. Two mechanisms, *Plasmodium* resistance and *Plasmodium* tolerance, are known to mediate the relationship between *Wolbachia* and *Plasmodium* parasites in mosquitoes [25]. The former reduces *Plasmodium* parasite load in the mosquitoes by two possible mechanisms, *Wolbachia*-mediated competition of the host resources [27, 28] or *Wolbachia*-mediated upregulation of the host immune system [29], although this has only been observed after novel *Wolbachia* infection in mosquitoes. The latter allows for high *Plasmodium* parasite load but with lower virulence in the mosquito by downregulation of the host immune response [25]. Both mechanisms, *Wolbachia*-induced *Plasmodium* resistance and tolerance, are believed to protect mosquitoes from *Plasmodium*-induced mortality [25]. However, the same mechanisms could also determine the natural prevalence of *Wolbachia* and *Plasmodium* co-infections in wild malaria vectors. The negative correlation between *Wolbachia* and *P. falciparum* in malaria vectors observed in Burkina Faso and Mali [38, 51] suggests *Plasmodium* resistance in *Wolbachia*-infected mosquitoes. Similarly, the low prevalence of *Wolbachia* and *P. falciparum* co-infection in *An. funestus* s.l. in southeastern Tanzania may be due to *Wolbachia*-induced resistance of the mosquitoes to *P. falciparum* infections. However, this observation may be limited by the fact that the prevalence of *P. falciparum* infection in *Wolbachia* uninfected mosquitoes was also very low (prevalence = 0.5%, *N* = 400), and indeed the sporozoite rate in *An. funestus* s.l. in this study was generally low (prevalence = 0.8%, *N* = 400). This poses a challenge in pinpointing the role of the detected *Wolbachia* strain on the low prevalence of *P. falciparum* infection in either group, *Wolbachia*-infected and *Wolbachia*-uninfected *An. funestus* s.l. Further studies are therefore required to ascertain the role of the indigenous *Wolbachia* strain in wild *An. funestus* s.l. in southeastern Tanzania on *P. falciparum* development, and should, perhaps, consider a larger sampling area and seasonality.

## Conclusions

This study provides the first report of natural *Wolbachia* infection and its phylogenetic characterization in *An. funestus* s.l., a major malaria vector in southeastern Tanzania. The low prevalence of *P. falciparum* infections and co-infections observed suggest that *Wolbachia* may influence parasite development, although the evidence remains inconclusive due to the small number of positive mosquitoes. The detection of strains clustering consistently within supergroup B highlights the ability of *An. funestus* s.l. to sustain stable *Wolbachia* infections in the field, distinct from previously reported patterns in *An. arabiensis*. While these findings suggest opportunities for *Wolbachia*-based strategies against insecticide-resistant and anthropophilic *An. funestus*, further large-scale and longitudinal studies are needed to clarify their role in malaria transmission and assess feasibility for control.

## Data Availability

Data supporting the main conclusions of this study are included in the manuscript.

## Abbreviations

bp: Base pairs
CDC: Centers for Disease Control and Prevention
*coxA*: Cytochrome c oxidase assembly
DNA: Deoxyribonucleic acid
ELISA: Enzyme-linked immunosorbent assay
FWD: Forward
GTR + G: General time-reversible plus Gamma distribution
IRS: Indoor residual spraying
LLINs: Long-lasting insecticidal nets
MLST: Multilocus strain typing
PCR: Polymerase chain reaction
rDNA: Ribosomal deoxyribonucleic acid
REV: Reverse
s.l.: Sensu lato
s.s.: Sensu stricto
TE buffer: Tris-EDTA buffer
*w*Anga_TZ: *Wolbachia Anopheles gambiae*_Tanzania
wsp: *Wolbachia* surface protein
